# Touchscreen Technology to Improve Dementia Care Management for Older Adults Living in the Community

**DOI:** 10.1093/geroni/igab046.3540

**Published:** 2021-12-17

**Authors:** Ellen Brown, Nicole Ruggiano, mariateresa H Munoz, lisa Roberts, C Victoria Framil, Bernardo Arroyo, Michelle Bourgeois

**Affiliations:** 1 Florida International University, Miami, Florida, United States; 2 University of Alabama, Hoover, Alabama, United States; 3 Florida International University, Florida International University, Florida, United States; 4 Unviersity of South Florida, Tampa, Florida, United States

## Abstract

Chronic disease management challenges for persons living with dementia (PLWD) often includes difficulties communicating how their feeling or describing symptoms to caregivers. To address this issue, an interdisciplinary team is developing a touchscreen mobile technology that will allow the assessment of clinical information about the PLWD, such as sleep quality, appetite, pain level, and mood. The mobile technology also allows caregivers to share this information with providers in real-time from their homes. In the team’s effort to incorporate a graphic measure to assess mood into the technology, a literature review revealed no such measurement tool has been established and validated for assessment with PLWD. This study focused on developing a graphic clinical assessment tool that can be used to assess mood for PLWD and establishing the face and content validity for this assessment tool. A team that included multidisciplinary clinical experts and a graphic artist designed a series of pictures of facial expressions and body language depicting a continuum of mood states (i.e., happy through depressed). The research team consulted with existing artistic depictions of sadness expression, including medical illustrations depicting depression. Multiple iterations have resulted in a series of images that represent moods from happy to very sad. The team considered a number of factors in developing the images including gender, ethnicity, and race. Consensus on the construct validity was achieved by the expert clinical panel. Details of a follow-up study to evaluate construct validity will be presented.

